# High Density Lipoprotein Protects Mesenchymal Stem Cells from Oxidative Stress-Induced Apoptosis *via* Activation of the PI3K/Akt Pathway and Suppression of Reactive Oxygen Species

**DOI:** 10.3390/ijms131217104

**Published:** 2012-12-13

**Authors:** Jianfeng Xu, Juying Qian, Xinxing Xie, Li Lin, Yunzeng Zou, Mingqiang Fu, Zheyong Huang, Guoping Zhang, Yangang Su, Junbo Ge

**Affiliations:** 1 Department of Cardiology, Zhongshan Hospital, Fudan University, Shanghai Institute of Cardiovascular Diseases, Shanghai 200032, China; E-Mails: doctorxjf@126.com (J.X.); novaxie@163.com (X.X.); linli777@126.com (L.L.); zou.yunzeng@zs-hospital.sh.cn (Y.Z.); barkfmq@yahoo.com.cn (M.F.); huang.zheyong@zs-hospital.sh.cn (Z.H.); su.yangang@zs-hospital.sh.cn (Y.S.); 2 Institutes of Biomedical Scienses, Fudan University, Shanghai 200032, China; E-Mail: gpzhang@fudan.edu.cn; 3 Department of Cardiology, Minhang Hospital, Ruijin Hospital Group, Shanghai Jiaotong University School of Medicine, Shanghai 201199, China

**Keywords:** high density lipoprotein, mesenchymal stem cells, oxidative stress, apoptosis, myocardial infarction

## Abstract

The therapeutic effect of transplantation of mesenchymal stem cells (MSCs) in myocardial infarction (MI) appears to be limited by poor cell viability in the injured tissue, which is a consequence of oxidative stress and pro-apoptotic factors. High density lipoprotein (HDL) reverses cholesterol transport and has anti-oxidative and anti-apoptotic properties. We, therefore, investigated whether HDL could protect MSCs from oxidative stress-induced apoptosis. MSCs derived from the bone marrow of rats were pre-incubated with or without HDL, and then were exposed to hydrogen peroxide (H_2_O_2_) *in vitro,* or were transplanted into experimentally infarcted hearts of rats *in vivo*. Pre-incubation of MSCs with HDL increased cell viability, reduced apoptotic indices and resulted in parallel decreases in reactive oxygen species (ROS) in comparison with control MSCs. Each of the beneficial effects of HDL on MSCs was attenuated by inhibiting the PI3K/Akt pathway. Preconditioning with HDL resulted in higher MSC survival rates, improved cardiac remodeling and better myocardial function than in the MSC control group. Collectively, these results suggest that HDL may protect against H_2_O_2_-induced apoptosis in MSCs through activation of a PI3K/Akt pathway, and by suppressing the production of ROS.

## 1. Introduction

An increasing number of studies have described transplantation of mesenchymal stem cells (MSCs) from bone marrow as a strategy for cardiac repair following myocardial infarction [1]. However, the therapeutic efficacy of this procedure is greatly limited by the poor survival of donor MSCs in the infarcted heart [2]. The injured myocardium represents an environment in which there are various factors that promote cell apoptosis, such as oxidative stress, hypoxia and inflammatory reactions [3]. Oxidative stress has been shown to be one of the key pro-apoptotic factors as it is present in both the ischemic stage and also during the reperfusion period [4,5]. Oxidative stress results in excessive accumulation of reactive oxygen species (ROS), which directly damages cell membranes, protein and DNA, thereby promoting cell senescence, compromising cell function and threatening cell survival [4,6]. Enhancing the viability of implanted MSCs and restoring cellular repair mechanisms are therefore critical factors in obtaining satisfactory outcomes with MSC-based therapy.

It is widely reported that high density lipoprotein (HDL) lowers the risks associated with ischemic cardiovascular diseases [7]. HDL has been reported to possess a variety of novel and functional properties in addition to its ability to reverse cholesterol transport (RCT) [8]. We have previously demonstrated that HDL abrogates cardiac hypertrophy induced by angiotensin II by down-regulating the expression of angiotensin II type 1 (AT-1) receptors [9]. Other workers have shown that the effects of experimentally induced ROS in the vascular wall were completely abolished by a daily infusion of reconstituted HDL [10]. HDL has also been shown to protect endothelial cells from primary apoptosis, and to reduce intracellular ROS induced by oxidized low-density lipoproteins [11]. These data suggested that HDL might have anti-oxidative and anti-apoptotic effects in cardiovascular pathophysiology.

We, therefore, investigated whether HDL could protect MSCs against oxidative stress-induced apoptosis. Additionally, we also examine the effect of HDL on the MSCs senescence, which was regarded to be induced by oxidative stress and to influence cell therapeutic potential considerably. Previously, the effects of HDL on endothelial progenitor cells [12], embryonic stem cells [13], hematopoietic stem cells [14] and induced pluripotent stem cells [13] have been reported, but to date there are no data describing the influence of HDL in the biological activity of MSCs. In the present study, we testified the impact of preconditioning with HDL on MSCs apoptosis and senescence induced by oxidative stress. In addition, hydrogen peroxide (H_2_O_2_), which is widely used as an oxidant [4,15], was applied *in vitro* to induce a form of cellular damage similar to oxidative stress.

## 2. Results and Discussion

### 2.1. HDL Protects MSCs against H_2_O_2_-Induced Apoptosis

**Figure 1:**
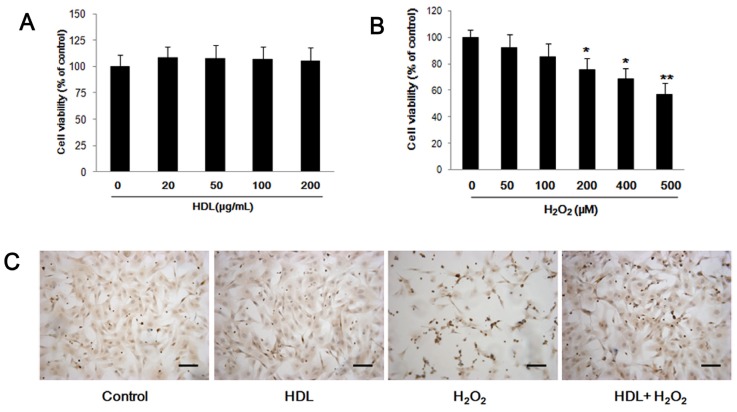

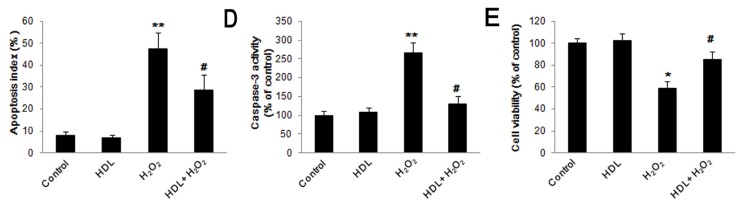
Effects of H_2_O_2_ and/or high density lipoprotein (HDL) on mesenchymal stem cell (MSC) viability and apoptosis. (**A**, **B**) MSCs were exposed to increasing concentrations of HDL (0 to 200 μg/mL) or H_2_O_2_ (0 to 500 μM) for 24 h; and cell viability was measured by MTS assay; (**A**) HDL did not significantly affect MSC viability; (**B**) H_2_O_2_ decreased the viability of MSCs in a concentration-dependent manner; (**C** to **E**) Cells were incubated with H_2_O_2_ (400 μM) following preconditioning with or without HDL (100 μg/mL) for 24 h. Cell apoptosis was measured by TUNEL and caspase-3 assays as shown in photomicrograph **C** (scale bar = 20 μm) and in histogram **D** respectively. MSC viability (**E**) was measured by MTS assay. Results were confirmed in three, independent experiments. *p < 0.05 *vs.* Control, **p < 0.01 *vs.* Control; # *p* < 0.05 *vs.* H_2_O_2_ group.

To investigate the effect of HDL on the biological activity of MSCs, we performed flow cytometry and MTS to detect cell differentiation and cell viability respectively. We observed that the MSCs (Passage 4), used in our experiments, expressed typical MSC-related cell surface antigens, which were positive for CD29, CD90 and negative for CD45, CD34; and that the incubation with HDL (100 μg/mL) for 24 h did not exhibit a notably impact on the characteristic phenotypes of MSCs (Figure S1). Meanwhile, we discovered that MSCs viability was not significantly affected by treatment with any of the tested concentrations of HDL (Figure 1A). Therefore, we chose a HDL concentration of 100 μg/mL for subsequent experiments based on methods described in previously published studies [9].

To determine the concentration of H_2_O_2_, which was employed to induce the cell apoptosis, MSCs was cultured with different concentration of H_2_O_2_ for 24 h and a MTS assay was performed afterwards. It was found that H_2_O_2_ impaired the viability of MSCs in a concentration-dependent manner over the tested concentration range (Figure 1B). The effect was statistically significant at concentrations over 200 μM. Based on these findings, 400 μM H_2_O_2_ was selected for use in the subsequent experiments.

In order to investigate the effects of HDL on H_2_O_2_-stimulated MSC apoptosis, MSCs were pretreated with or without HDL (100 μg/mL) for 24 h and then cultured with 400 μM of H_2_O_2_ for an additional 24 h. TUNEL assay showed that incubation with 100 μg/mL HDL did not affect the apoptosis of MSCs (HDL group *vs.* Control group: (6.83 ± 1.35)% *vs.* (7.93 ± 1.76)%, *p* > 0.05). However, H_2_O_2_ significantly increased MSCs apoptosis (H_2_O_2_ group *vs.* Control group: (47.37 ± 7.53)% *vs.* (7.93 ± 1.76)%, *p* < 0.01), which was remarkably attenuated after preconditioning with HDL (HDL + H_2_O_2_ group *vs.* H_2_O_2_ group: (28.77 ± 6.91)% *vs.* (47.37 ± 7.53)%, *p* < 0.05) (Figure 1C). Caspase-3 activity was significantly increased in H_2_O_2_-stimulated MSCs compared to controls ((267.4 ± 25.3)% *vs.* (100 ± 12.2)%, *p* < 0.01). The increased caspase-3 activity was ameliorated by pre-incubation with HDL (HDL + H_2_O_2_ group *vs.* H_2_O_2_ group: (130.9 ± 19.7)% *vs.* (267.4 ± 25.3)%, *p* < 0.05) (Figure 1D). Similarly, compared to H_2_O_2_ group, HDL pretreatment restored cell viability following exposure to H_2_O_2_ (HDL + H_2_O_2_ group *vs.* H_2_O_2_ group: (85.3 ± 7.2)% *vs.* (58.6 ± 6.8)%, *p* < 0.05) (Figure 1E).

**Figure 2:**
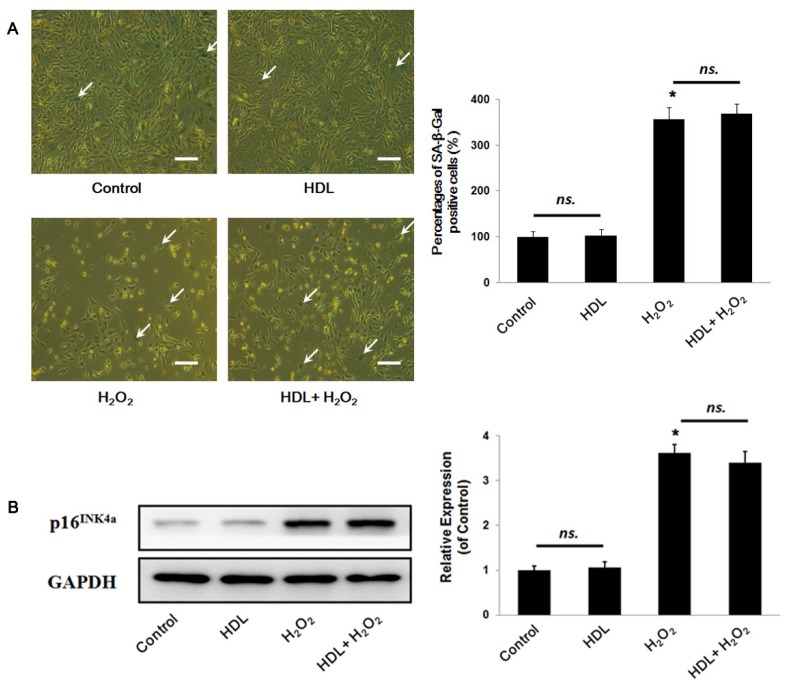
Influence of HDL in the MSC senescence. MSCs were treated with or without HDL for 24 h followed by exposure to H_2_O_2_ insult for another 24 h or not; (**A**) Senescence-associated β-Galactosidase staining and representative photograms from three independent experiments are displayed (scale bar = 20 μm); (**B**) The expressions of p16^INK4a^ were detected by Western blot analysis and representative bands are shown. Data are shown as mean ± SE from three independent experiments. *p < 0.05 *vs.* Control group, *ns.*, not significant; GAPDH, glyceraldehyde 3-phosphate dehydrogenase.

Meanwhile, MSCs aging or senescence, which directly impaired the regenerative capability, was widely reported to be induced by oxidative stress [16,17]. We, therefore, wondered whether HDL treatment could protect MSCs against H_2_O_2_-stimulated replicative exhaustion. Senescence-associated β-Galactosidase (SA-β-Gal) staining showed that there were no significant differences in the percentages of SA-β-Gal positive cells between the HDL group and the Control group ((102.3 ± 13.7)% *vs.* (100 ± 12.4)%, *p* > 0.05). Similarly, there was nearly identical in those between the H_2_O_2_ group and the HDL+H_2_O_2_ group ((356.2 ± 25.8)% *vs.* (367.9 ± 22.37)%, *p* > 0.05), although there was a significant increment compared the H_2_O_2_ group with the Control one (*p* < 0.05) (Figure 2A). Furthermore, the MSCs senescence was confirmed by Western blot assay, suggesting that there were no remarkably differences in the expression of p16^INK4a^ between the HDL group and the Control ((1.06 ± 0.13) *vs.* (1.00 ± 0.10), *p* > 0.05), and between the H_2_O_2_ group and the HDL + H_2_O_2_ group ((3.62 ± 0.19) *vs.* (3.40 ± 0.26), *p* > 0.05). Likewise, it was found that the expression of p16^INK4a^ in the H_2_O_2_ group statistically increased than that in the Control one (*p* < 0.05) (Figure 2B). Therefore, it indicated that HDL exerted no statistical effects on the senescence of MSCs both under a condition of oxidative stress and under that of control.

### 2.2. PI3K/Akt Pathway Involves in the Protective Effect of HDL

**Figure 3:**
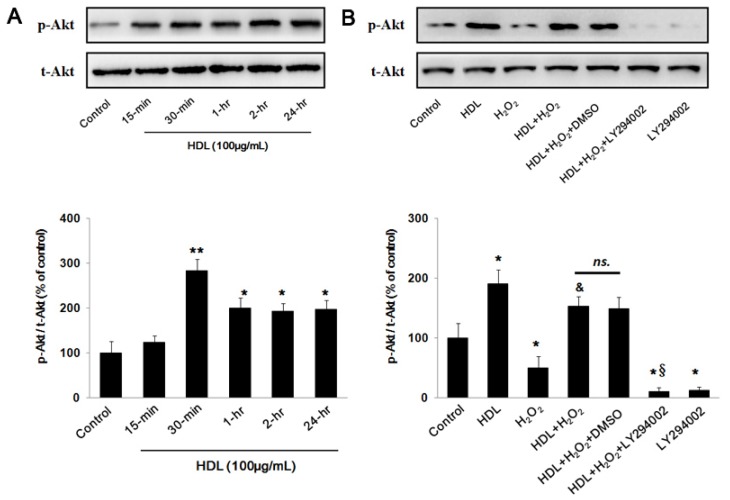

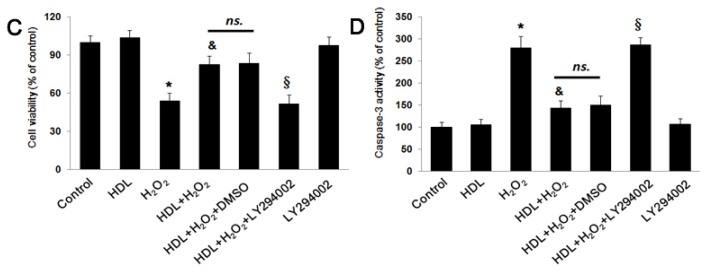
Role of PI3K/Akt pathway in the HDL induced anti-apoptotic protection. (**A**) Western blot analysis show that HDL significantly increased Akt phosphorylation in MSCs. (**B** to **D**) MSCs were pre-incubated with LY294002 (25 μM) or DMSO for 1 h and then exposed to HDL (100 μg/mL) followed by exposure to H_2_O_2_ (400 μM) for a further 24 h. Western blotting results show that Akt phosphorylation induced by HDL was decreased by pre-incubation with LY294002. The protective effect of HDL evidenced by cell viability and caspase-3 activity was significantly abolished by LY294002. Results were confirmed in three, independent experiments. *p < 0.05 *vs.* Control, **p < 0.01 *vs.* Control, and *p* < 0.05 *vs.* H_2_O_2_ group, § *p* < 0.05 *vs.* HDL + H_2_O_2_ + DMSO group, *ns.*, not significant; DMSO, Dimethyl sulfoxide.

As previously reported [18], PI3K/Akt pathway played a critical role in the cell apoptosis. To examine our hypothesis that PI3K/Akt pathway was involved in the protective effect of HDL on MSCs, Western blot analysis was used to assess the changes in Akt phosphorylation in MSCs exposed to HDL with different concentration. The results indicated that incubation with HDL significantly up-regulated Akt phosphorylation. This response began 15 min after exposure, peaked at approximately 30 min (30-min group *vs.* Control group: (283.8 ± 25.1)% *vs.* (100 ± 24.71)%, *p* < 0.05) and was still present at 24 h (24-h group *vs.* Control group: (197.5 ± 19.37)% *vs.* (100 ± 24.71)%, *p* < 0.05) (Figure 3A), suggesting that HDL could enhance the activation of PI3K/Akt pathway in MSCs. To provide further evidence, a PI3K inhibitor, LY294002, was effectively employed to decrease Akt activation (LY294002 group *vs.* Control group: (12.37 ± 5.19)% *vs.* (100 ± 24.28)%, *p* < 0.05) (Figure 3B). Considering that high concentrations of LY294002 could cause a reduction in cell viability or a generation of ROS, a LY294002 group was added to examine its toxicity to MSCs. It was observed that LY294002, at a concentration of 25 μM, did not exhibit a statistical effect on the MSCs viability (LY294002 group *vs.* Control group: (97.6 ± 6.8)% *vs.* (100 ± 5.1)%, *p* > 0.05) (Figure 3C) and apoptosis (LY294002 group *vs.* Control group: (106.4 ± 13.5)% *vs.* (100 ± 11.3)%, *p* > 0.05) (Figure 3D), which was consistent with what was testified previously [19]. Intriguingly, the pre-treatment of LY294002 at this concentration was found to abolish the protective effect of HDL on H_2_O_2_-stimulated cells. This resulted in decreased cell viability as measured by MTS assay (HDL + H_2_O_2_ + LY294002 group *vs.* HDL + H_2_O_2_ + DMSO group: (51.7 ± 7.1)% *vs.* (83.6 ± 7.8)%, *p* < 0.05) (Figure 3C) and increased apoptosis estimated by changes in caspase-3 activity (HDL + H_2_O_2_ + LY294002 group *vs.* HDL + H_2_O_2_ + DMSO group: (286.3 ± 16.7)% *vs.* (149.5 ± 21.8)%, *p* < 0.05) (Figure 3D), consistently indicating that HDL protected MSCs against injury induced by H_2_O_2_ through PI3K/Akt pathway.

### 2.3. Phosphorylation of Akt Induced by HDL Inhibits ROS Generation

**Figure 4:**
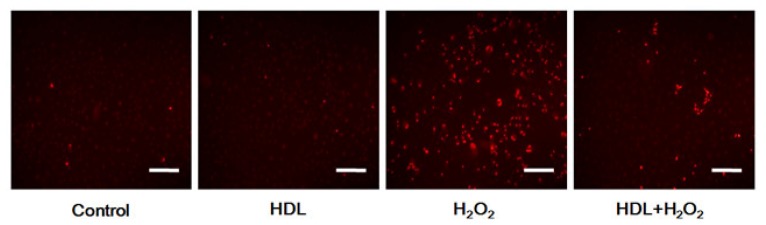

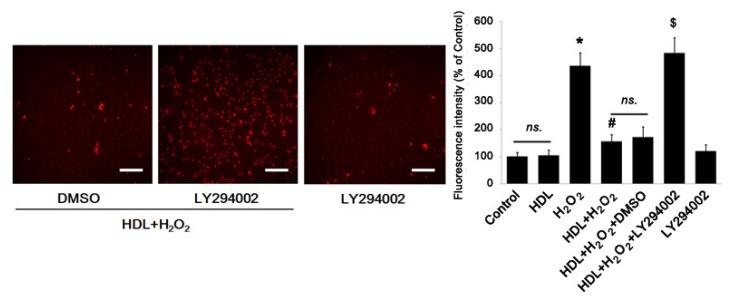
Impact of activation of PI3K/Akt pathway induced by HDL on reactive oxygen species (ROS) generation. The intracellular ROS in MSCs was visualized by fluorescent microscopy. Representative photomicrographs (scale bar = 100 μm) and corresponding histograms are displayed. Preconditioning with HDL inhibited H_2_O_2_-stimulated ROS generation in MSCs. However, the protective effect disappeared with LY294002 pretreatment. Results were confirmed in three independent experiments. *p < 0.05 *vs.* Control group, # *p* < 0.05 *vs.* H_2_O_2_ group, $ *p* < 0.05 *vs.* HDL + H_2_O_2_ + DMSO group, *ns.*, not significant; DMSO, Dimethyl sulfoxide.

The influence of the PI3K/Akt pathway in ROS production based on LY294002 inhibition and assessed using fluorescent probe-DHE demonstrated that the percentage of ROS negative cells with low fluorescence intensity were similarly observed among the Control, the HDL and the LY294002 groups (all *p >* 0.05) (Figure 4). Cells with strong red fluorescent, indicating ROS accumulation, were more prevalent in the H_2_O_2_ group than in the Control one ((435 ± 48)% *vs.* (100 ± 15)%, *p* < 0.05). HDL pretreatment restored intracellular ROS generation induced by H_2_O_2_ (HDL + H_2_O_2_ group *vs.* H_2_O_2_ group: (156 ± 25)% *vs.* (435 ± 48)%, *p* < 0.05), and the protective effect disappeared when the PI3K/Akt pathway was inhibited by LY294002 (HDL + H_2_O_2_ + DMSO group *vs.* HDL + H_2_O_2_ + LY294002 group: (172 ± 37)% *vs.* (482 ± 57)%, *p* < 0.01).

The above results showed that the inhibition of PI3K/Akt pathway attenuated the protective effect of HDL and the decreased ROS, indicating that Akt activation was partly upstream of ROS generation. Excessive production of ROS has been shown to damage stem cells through caspase-mediated pathways [20]. Therefore, these findings suggested that HDL activation of the PI3K/Akt pathway might inhibit ROS generation, and thereby reduce apoptosis of implanted MSCs.

### 2.4. HDL Pre-Incubation Promotes MSC Survival in the Infarcted Heart

**Figure 5:**
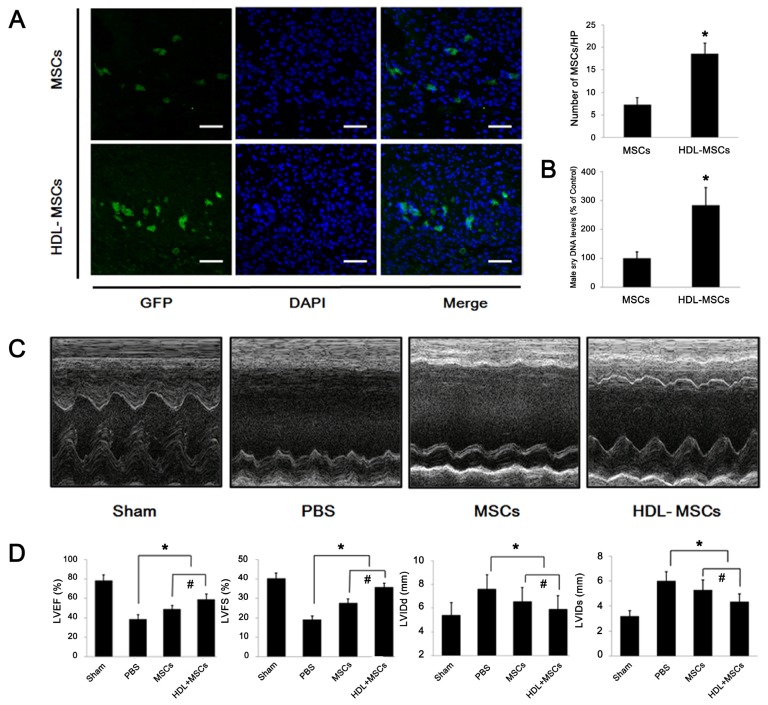
Effects of HDL preconditioning on transplanted MSCs survival and cardiac function in the rats with experimental MI. (**A** and **B**) The number of GFP^+^ MCSs was counted under fluorescent microscopy four days after injection. Representative photomicrographs (**A**, scale bar = 100 μm) and the corresponding histogram are displayed. Male *sry* DNA was examined by real-time PCR. *n* = 7 per group. *p < 0.05 *vs.* MSCs group. (**C** and **D**) four weeks after therapeutic intervention, transthoracic echocardiography was performed to evaluate cardiac remodeling and LV function. Representative M-mode echocardiograms and quantitative data are shown. *n* = 8 per group. * *p* < 0.05 *vs.* PBS group, # *p* < 0.05 *vs.* MSCs group.

The poor viability of the donor cell, typically <1% at 4 days after transplantation, has been reported in the previous study regarding MSCs-base therapy [5]. Therefore, we testified the effect of HDL preconditioning on the survival of MSCs on Day 4 after transplantation *in vivo* (Figure 5A,B). After a GFP-labeling and the pre-incubation with or without HDL (100 μg/mL), MSCs (1 × 10^6^ in 100 μL) were respectively transplanted into the infarcted heart of rats. It was discovered that the number of GFP^+^ MSCs per HP in the HDL-MSCs group was 2.58-fold greater than that in the MSCs group (*p* < 0.05). Furthermore, when we transplanted the male MSCs to the female hearts, it was revealed that the *sry* DNA level in the HDL-MSCs group was approximately 2.83-fold higher than that in the MSCs group (*p* < 0.05), indicating that HDL preconditioning could enhance the survival of MSCs after transplantation.

### 2.5. MSCs Preconditioned with HDL Reduce Cardiac Remodeling and Preserve Myocardial Function

The effect of MSCs preconditioned with HDL on cardiac function was observed by transthoracic echocardiography four weeks after experimentally induced MI in rats (Figure 5C,D). It indicated that left ventricular ejection fraction (LVEF) was 50.8% lower in the PBS group than in the Sham group and LVFS was 53.0% lower in the PBS group than in the Sham group (both *p* < 0.05); and that LVIDd was 40.9% higher and LVIDs was 90.0% higher in the PBS than in the Sham group (both *p* < 0.05). Furthermore, compared with PBS group, transplantation of MSCs improved LVEF and LVFS by 13.0% and 21.6%, respectively (both *p* < 0.05). Injection of HDL-MSCs enhanced the LVEF and LVFS by an additional 13.0% and 20.1%, respectively (both *p* < 0.05). Transplantation of MSCs decreased the LVIDd and LVIDs by 19.5% and 23.3%, respectively compared to the PBS group (both *p* < 0.05), and transplantation of HDL-MSCs further decreased LVIDd and LVIDs by approximately 12.3% and 30.0% (both *p* < 0.05), respectively.

We noticed that Kiya Y *et al.*[21] had previously reported that continual infusion of HDL following MI prevented LV remodeling and improved myocardial function in rats. Though they attributed the better prognosis to an improvement of the survival of cardiomyocytes resulting from treatment with HDL, they had not well explored the influence of HDL on the cells that migrated to injured myocardium, such as MSCs. And our results from the present study might partly provided the evidences on a novel perspective to support their finding as follows: a higher level of HDL might improve the anti-apoptotic capability of MSCs, which were mobilized to the damaged myocardial region, thus contributing to the myocardial regeneration, bringing about a better cardiac function after MI.

In the recent literature, the effect of transplantation of MSCs on myocardial repair was reported as confined for the low viability of graft cells [1,22]. For instance, even 6 × 10^7^ implanted MSCs in infarcted porcine hearts yielded only limited enhancement of LVEF [23]. A series of pharmacological preconditioning was thought to be an effective approach for promoting MSCs survival [24] but had the potential to cause severe side effects in patients with cardiovascular diseases.

Therefore, we explored whether improving the in-situ physiological environment of MSCs could enhance cells viability and prevent oxidative stress-induced apoptosis after transplantation or mobilization. Epidemiological studies have consistently shown an inverse correlation between HDL levels and outcomes in ischemic heart diseases [8]. We endeavored to elucidate this correlation with a hypothesis that HDL might promote survival of implanted MSCs, ameliorate their capacity to repair damaged tissue and thereby improve clinical prognosis. In support of this hypothesis, our data indicated that HDL protected MSCs from apoptosis induced by H_2_O_2_*in vitro.* The mechanisms involved activation of PI3K/Akt pathway, which was consistent with findings from previously reported studies [15,22]. We also demonstrated that the anti-apoptotic effect of HDL enhanced survival of MSCs and improved myocardial repair *in vivo*, supporting the findings of previous researches showing that the administration of HDL preserved the post-ischemic cardiac function [25].

Beside apoptosis, cellular senescence had been proved to be the other main outcome following oxidative stress stimuli [16], which considerably impaired MSCs therapeutical properties. On the other hand, there were several literature suggesting that a higher level of HDL was associated with a slower rate of leukocyte telomere length shortening per year [26]; and that offspring of individuals with exceptional longevity had significantly larger HDL, which hinted that HDL was probably involved in the cellular aging [27]. Considering those evidences, we carried out the parallel experiments to investigate whether HDL could play a critical role in the protective effect on MSCs senescence stimulated by oxidative stress. H_2_O_2_ treatment expectably spurred a considerable replicative exhaustion in MSCs; however, MSCs senescence could not be reversed by HDL preconditioning, which indicated that the effectiveness of HDL on the MSCs therapeutical potential *in vivo* was independent on the influence in cellular aging, while mainly through enhancement in cellular anti-apoptotic property. Indeed, the elucidation of this mechanism is especially critical for physicians engaged in cell-based therapy, whom must be sure that MSCs are devoid of chromosome alterations and that there are no signs of cellular senescence.

The clinical significance of our results is the possible benefit of HDL in patients with myocardial infarction in terms of improving the survival of transplanted MSCs. Elevation of HDL levels in patients, at high risk of cardiovascular disease may also be of vital clinical importance in preventing cardiovascular events. However, we did not identify the specific components of HDL that were responsible for its anti-oxidant and anti-apoptotic effects in the present study, for HDL is a lipoprotein, containing HDL2 and HDL3, with a density ranged from 1.063 to 1.210 g/mL [28]. This will be the subject of future research, along with an investigation of other potential effects of HDL on MSCs (such as promotion of secretion); that may contribute to the restoration of cardiac function *in vivo*.

## 3. Experimental Section

### 3.1. Isolation and Culture of MSCs

All animal procedures were undertaken in accordance with the Guidelines for the Care and Use of Laboratory Animals, published by the National Academy Press (NIH Publication No. 85–23, Revised 1996). The experiments were approved by the Animal Care and Use Committee of Zhongshan Hospital, Fudan University.

MSCs were isolated from four-week-old Sprague-Dawley (SD) rats weighing 100 to 120 g by flushing femurs and tibias with Dulbecco’s modified Eagle’s medium (DMEM; GIBCO), 10% Fetal Bovine Serum (FBS; GIBCO) and penicillin/streptomycin. The bone marrow (BM) solution was cultured at 37 °C in an atmosphere of 5% CO_2_. The culture medium was changed every 3–4 days. Non-adherent hematopoietic cells were removed during this process, and the adherent, spindle-shaped MSCs were cultured and expanded. Passage 4 cells were used for subsequent experiments.

### 3.2. Adenoviral Transduction and Labeling of MSCs

An adenoviral vector carrying a green fluorescent protein (GFP) (GeneChem) for cell transduction was used to establish and identify a stable population of MSCs. Transduction was facilitated by transfecting Passage 4 MSCs with purified adenovirus cells that had been exposed to 7 μg/mL polybrene. The transfected cells were incubated for 24 h at 37 °C with 5% CO_2_. The medium was removed, replaced with fresh medium and incubated for a further 24 h. Cells were then sorted by flow cytometry for GFP to ensure that a homogenous population of GFP^+^ MSCs was obtained (data were not shown).

### 3.3. *In vitro* Exposure to H_2_O_2_ and HDL

MSCs were incubated for 6 h with DMEM supplemented with 1% FBS prior to experimental interventions. The starved MSCs were then respectively exposed to H_2_O_2_ (0 to 500 μM) or HDL (0 to 200 μg/mL; Merck) for 24 h. The experiments were undertaken in four groups: a control group; a HDL group (MSCs incubated with HDL 100 μg/mL for 24 h); a H_2_O_2_ group (incubation with 400 μM H_2_O_2_ for 24 h); a HDL + H_2_O_2_ group (pre-incubation with HDL 100 μg/mL for 24 h, followed by 24-h exposure to 400 μM H_2_O_2_). For inhibitor experiments, three additional groups were set as follows: MSCs were pre-incubated with PI3K inhibitor (LY294002, 25 μM) or vehicle (DMSO) for 1 h, and then successively treated with HDL and H_2_O_2_ as above; the third group was pre-cultured with LY294002 alone. The concentrations of HDL, H_2_O_2_ and LY294002 used in these experiments were determined from pilot studies and based on similar methods described in the literature [9,19,29].

### 3.4. MSC Viability Assay

MSC viability was assessed using a 3-(4,5-dimethylthiazol-2-yl)-5-(3-carboxymethoxyphenyl)-2- (4-sulfophenyl)-2*H*-tetrazolium, inner salt (MTS) assay. Cells were cultured in 24-well plates and treated according to the protocol. After 24 h, Cell Titer 96^®^ Aqueous One Solution Reagent (Promega) was added to each well according to the manufacturer’s instructions. Cell viability was evaluated after 2-h incubation with this reagent, by testing absorbance at 490 nm using a plate reader.

### 3.5. TUNEL (Terminal Dexynucleotidyl Transferase-Mediated dUTP Nick end Labeling) Assay

Cell apoptosis was investigated using an In Situ Cell Death Detection kit (Roche). In brief, MSCs were fixed in 3.7% buffered formaldehyde, pretreated with 3.0% H_2_O_2_ and exposed to TdT enzyme at 37 °C for 1 h. The cells were then incubated with digoxigenin-conjugated nucleotide substrate at 37 °C for 30 min. Nuclei exhibiting DNA fragmentation were identified after staining with 3,3-diamino benzidine (DAB) for 5 min.

The MSCs were counterstained with hematoxylin and viewed by light microscopy. Five fields at high magnification (×200) were randomly chosen to assess the apoptosis index, which was calculated as a percentage of labeled cells to total nuclei.

### 3.6. Caspase-3 Activity Assay

Caspase-3 activity was determined in MSC lysates (200 μg) using Caspase-3/CPP32 Fluorometric Assay kit (BioVision). The fluorogenic CPP32/caspase-3 substrate was labeled with the fluorochrome 7-amino-4-methyl coumarin. In this assay, the amount of fluorescence produced upon cleavage was proportional to the amount of caspase-3 activity present in the sample.

### 3.7. Senescence-Associated β-Galactosidase Staining

MSCs cultured in six-well plates were rinsed with phosphate-buffered saline (PBS), fixed and then incubated with freshly prepared senescence-associated β-Galactosidase (SA-β-Gal) staining solution (Beyotime, China) at 37 °C overnight. Five fields at high magnification (×200) were randomly chosen to count the number of blue cells (SA-β-Gal positive cells). At least 500 cells in each group were examined and the percentages of blue cells presented.

### 3.8. Western Blot Analysis

MSCs from each group were rinsed with cold PBS and lysed using RIPA buffer containing 1 mM phenylmethanesulfonylfluoride on ice for 30 min. The lysates were transferred to 1.5 mL Eppendorf tubes and centrifuged at 4 °C and 15,000 rpm for 30 min. The protein component was separated by 12% SDS-PAGE and then transferred to polyvinylidene fluoride membranes (Millipore). The membranes were blocked in 5% skimmed milk for 2 h at room temperature, and were incubated overnight at 4 °C with anti-phospho-Akt (Cell Signaling), anti-Akt (Cell Signaling), anti-p16^INK4a^. Following a 30 min wash, the membranes were incubated for 1 h with a secondary antibody conjugated to horseradish peroxidase-conjugated IgG (Jackson). Protein expression was determined using an enhanced chemiluminescence system and quantified by densitometry (Image System; Bio-Rad).

### 3.9. Measurement of ROS

The production of intracellular ROS was measured using an oxidation-sensitive fluorescent dihydroethidium (DHE) probe (Vigorous). The assay was based on the principle that DHE crosses cell membranes and is rapidly oxidized in the presence of ROS resulting in the formation of a highly fluorescent form of oxidative ethidium.

In this assay MSCs were washed three-times with PBS and incubated with 10 μM DHE in phenol-red-free MEM medium (Invitrogen) at 37 °C in the dark. After 15 min incubation, the cells were rinsed and the medium was replaced. The fluorescence level, as an indicator of ROS production, was detected using fluorescence microscopy. The excitation and emission filters were 488 nm and 590 nm, respectively.

### 3.10. Myocardial Infarction and MSCs Transplantation

The MI model was developed in SD rats (220 ± 30 g), as previously described [22]. MSCs (1 × 10^6^ in 100 μL, derived from male rats) (MSCs group) or MSCs preconditioned with HDL (HDL-MSCs group) were intramyocardially injected into female rats 10 min after ligation-induced infarction. The MSCs were injected into 3 to 5 injection sites into the anterior aspect of the viable myocardium that bordered the area of infarction. For the experiments concerning cardiac remodeling, two additional groups were set as follows: rats received intramyocardial injection of 100 μL of PBS without MSCs (PBS group) or received thoracotomy only but were not ligated (Sham group).

### 3.11. Tracking of the GFP^+^ MSCs Injected in MI Hearts

To observe the donor MSCs survival *in vivo*, rats were sacrificed four days after cell implantation. Hearts were harvested and sliced transversally above the ligation suture. Tissue fixation was minimized to reduce autofluorescence. The sample was embedded at optimum cutting temperature and cut by a cryostat from the base (above the ligation) towards the apex until the ligation suture was reached. Beginning at this point, 10 μm sections were collected throughout the entire lesion. The sections were stained using DAPI (4,6-diamidino-2-phenylindole), and the number of GFP^+^ MSCs was estimated under fluorescent microscopy at a magnification of ×200.

Three photomicrographs were taken from the infarct border zone of each section. Six sections were selected for counting, giving a total of 18 high power fields per rat. The numbers of GFP^+^ MSCs were counted and averaged.

### 3.12. Real-Time Polymerase Chain Reaction

Real-time polymerase chain reaction (PCR) was performed for quantification of the male *sry* DNA levels on Day 4 after cell transplantation. Rats were sacrificed and hearts were excised, frozen in liquid nitrogen and powdered. DNA purification was performed using Genomic DNA Isolation kit (Qiagen). The concentration of the purified DNA was determined by spectrophotometry. Real-time PCR was performed using Takara SYBR Premix Ex TaqTM in a Bio-Rad iQ5 optical module. Primers for amplification of rat Y chromosome *sry* and *β-actin* genes were synthesized (Invitrogen) and were listed as follows: *sry*, forward, 5′-GAG GCA CAA GTT GGC TCA ACA-3′; reverse, 5′-CTC CTG CAA AAA GGG CCT TT-3′. *β-actin*, forward, 5′-CCA TTG AAC ACG GCA TTG-3′; reverse, 5′-TAC GAC CAG AGG CAT ACA-3′. The cycling conditions were: 30 s at 95 °C, followed by 40 cycles of 5 s at 95 °C, 30 s at 59.5 °C, 30 s at 72 °C. Melting curves were acquired at the end of the reaction by gradually raising the temperature by 1 °C/min from 59.5 °C to 95 °C over a time period of 35 min.

### 3.13. Echocardiography

Cardiac remodeling and left ventricular function were assessed by transthoracic echocardiography 4 weeks after MI, using a Vevo 770 high-resolution imaging system (Visual Sonics) with a 17.5-MHz probe. After the induction of light general anesthesia, hearts were imaged in two dimensional and M-modes. The recordings were obtained in the para-sternal long-axis view at the level of the greatest LV diameter. The LV internal end-diastolic diameter (LVIDd) and LV internal end-systolic diameter (LVIDs) were measured from M-mode recordings according to the leading-edge method. All echocardiographic measurements were averaged from at least five separate cardiac cycles.

### 3.14. Statistical Analysis

Statistical analysis was undertaken using SPSS version 16.0. All data were expressed as means and standard errors (SE). Between-group of differences was analyzed using Student’s *t*-test. Comparisons between more than two groups were performed using one-way analysis of variance (ANOVA) with Bonferroni’s correction. Values of *p <* 0.05 were considered statistically significant.

## 4. Conclusions

In summary, we demonstrated that HDL protected MSCs from H_2_O_2_-induced apoptosis by activation of the PI3K/Akt pathway and down-regulation of ROS generation; and that preconditioning with HDL promoted MSCs survival after cell transplantation, thereby preserving cardiac function. This observation provides an experimental basis for improving the prognosis of patients with myocardial infarction through elevation of HDL levels. The HDL-based therapy may hold the promise of reducing the incidences of such left ventricular remodeling and heart failure after myocardial damage.

## Supplementary Materials
