# Supplementary material for: High Density Lipoprotein Protects Mesenchymal Stem Cells from Oxidative Stress-Induced Apoptosis via Activation of the PI3K/Akt Pathway and Suppression of Reactive Oxygen Species
Source: Int J Mol Sci. 2012 Dec 13;13(12):17104–20. doi: 10.3390/ijms131217104 (PMC3546741; doi:10.3390/ijms131217104)
Supplement: Supplementary file 1 [file ijms-13-17104-s001.pdf]

Supplementary Materials

1. Supplementary Method

Flow Cytometry

MSCs at passage 5 (P5), cultured with or without HDL (100 μg/mL) for 24 h, were harvested and washed twice with PBS containing 0.1% bovine serum albumin (BSA). 1 × 10^6 MSCs were respectively incubated with FITC-conjugated anti rat CD34 (BD pharmingen, USA), or PE-conjugated anti rat CD29 (BD pharmingen, USA), CD45 (BD Bioscience, USA) and CD90 (BD Bioscience, USA) at 4 °C for 30 min, and then washed twice with PBS containing 0.1% BSA. Isotype-identical antibodies (IgG) served as controls. The cells were analyzed by flow cytometric analysis using a Beckman Counter flow cytometer (USA) with the application of EXPO32 MultiCOMP software. Three independent experiments were performed and cells phenotypes were compared, with the mean level reported, between HDL-MSCs and MSCs.

2. Supplementary Result

The Influence of HDL on the Differentiation of MSCs

Flow cytometry analysis was performed to determine whether HDL treatment influenced the differentiation of MSCs. It revealed that the MSCs, derived from bone marrow of rats and used in our experiments, expressed typical MSC-related cell surface antigens, which were positive for CD29, CD90 and negative for CD45, CD34 (Figure S1). Furthermore, HDL presented no significant influence in the typical phenotypes of MSCs after 24-h incubation, which was indicated by the similar expression of surface antigens between MSCs group and HDL-MSCs group (CD34: (5.65 ± 0.57)% *vs.* (6.27 ± 0.72)%; CD45: (7.68 ± 0.68)% *vs.* (6.33 ± 0.77)%; CD29: (95.3 ± 2.35)% *vs.* (93.7 ± 3.48)%; CD90: (92.8 ± 3.27)% *vs.* (91.8 ± 2.87)%, all $p > 0.05$).

3. Supplementary Figure Legend

Figure S1. Flow cytometry demonstrated the cell phenotypes and the influence of HDL in the differentiation of MSCs. The typical MSC-related cell surface antigens in MSCs, cultured with HDL for 24 h or not, were examined. Data are shown as mean ± SE from three independent experiments.

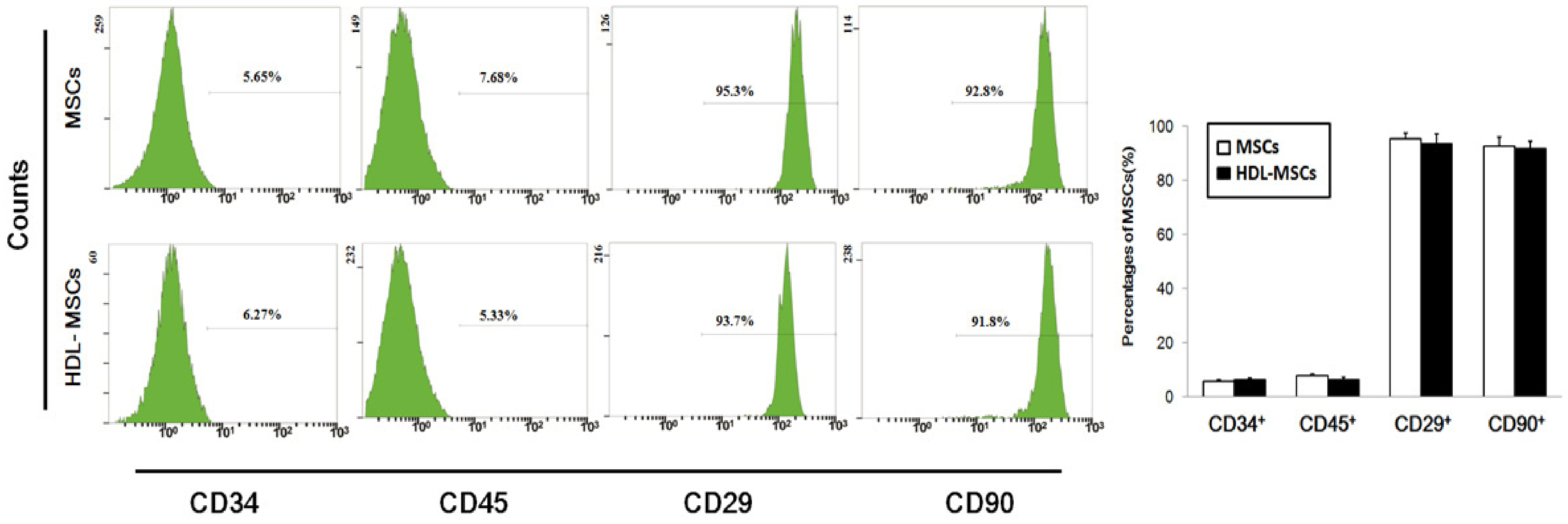

© 2012 by the authors; licensee MDPI, Basel, Switzerland. This article is an open access article distributed under the terms and conditions of the Creative Commons Attribution license (http://creativecommons.org/licenses/by/3.0/).
